# Preparing Europe for imported Andes virus: the role of rapid serology and molecular diagnostics

**DOI:** 10.1016/j.lanepe.2026.101724

**Published:** 2026-05-15

**Authors:** Thirumalaisamy P. Velavan, Jonas Schmidt-Chanasit

**Affiliations:** a Institute of Tropical Medicine, University of Tübingen, and German Center for Infection Research (DZIF), Tübingen, Germany; b Vietnamese German Center for Medical Research, Hanoi, Vietnam; c Faculty of Medicine, Duy Tan University, Da Nang, Vietnam; d Bernhard Nocht Institute for Tropical Medicine, Hamburg, Germany; e Faculty of Mathematics, Informatics and Natural Sciences, Universität Hamburg, Hamburg, Germany

The outbreak of Andes virus (ANDV) linked to the expedition ship *MV Hondius* has brought a rare New World hantavirus to European public health attention: as of 8 May 2026, WHO reported eight linked cases and three deaths, including six laboratory-confirmed and two probable infections, with hospitalizations in South Africa, the Netherlands, and Switzerland.^1^^,^^2^

The remaining passengers and crew members who have arrived in Spain have repatriated to their home countries under the supervision of the health authorities, thereby shifting the focus of the measures from an outbreak on the ship to cross-border contact tracing. Unlike Old World hantaviruses, which in Europe and Asia mainly cause haemorrhagic fever with renal syndrome (HFRS) or nephropathia epidemica, New World hantaviruses such as ANDV cause hantavirus cardiopulmonary syndrome (HCPS), a rapidly progressive disease with higher mortality.^3^ ANDV is also exceptional because, in addition to rodent-borne transmission, human-to-human spread is well documented, meaning that a suspected imported case in Europe may immediately require not only diagnosis and clinical management, but also isolation, contact tracing, and coordinated reference-laboratory confirmation.

For Europe, the priority should be a tiered diagnostic algorithm that links rapid local triage with definitive reference laboratory confirmation. Suspected cases should first be recognised based on compatible clinical symptoms and a careful exposure and travel history. Front-line laboratories should then have access to widely available commercial serological assays capable of detecting infections with both Old and New World hantaviruses. In addition, reliable rapid IgM/IgG tests would be particularly useful wherever an immediate decision is required before referral samples can reach a national reference laboratory. Such tests would not replace reference diagnostics, but could support early triage, infection-control decisions, clinical management, and prioritisation of confirmatory testing.

The practical challenge is not to choose between PCR and serology, but to enable rapid triage of a febrile traveller, a cruise passenger or contact, or a critically ill patient before samples reach a national reference laboratory. Molecular tests have an important role for confirming suspected ANDV infection. RT-qPCR assays have a high diagnostic performance with a low limit of detection, supporting its use as an early diagnostic tool.^4^ For ANDV, RT-qPCR can be informative during acute phase and recent prospective data indicate that viral RNA may remain detectable in many patients well beyond the first week of illness.^4^^,^^5^ At the same time, symptomatic patients with New World hantavirus infection are already IgM-positive at clinical presentation, making serology a central component of acute diagnosis.^6^

Serology remains central, because the antibody response is often detectable by the time ANDV patients become symptomatic, unlike many acute viral infections. In European Puumala hantavirus infection, IgM and IgG antibodies can be detected shortly after the disease onset, with the nucleocapsid protein being the primary target of the early antibody response.^7^ In HCPS, patients can develop IgM and IgG antibodies at an early stage, meaning that serology remains clinically meaningful once symptoms have appeared.^3^^,^^6^ This feature is crucial for outbreak triage, as an IgM/IgG-based test can already be informative when patients first present with fever, gastrointestinal symptoms, thrombocytopenia, dyspnoea or any evolving pulmonary edema.

A solid-phase enzyme immunoassay using ANDV recombinant nucleoprotein detected early, and strong IgM, IgG, and IgA responses and ANDV-specific IgM antibodies were found in all patients in the first available sample and remained detectable for at least 43 days.^6^ Further analysis demonstrated high sensitivity and specificity for IgM and IgG ELISAs.^3^ Multiparametric immunofluorescence assays using antigens from Old World and New World hantaviruses have achieved high sensitivity, including a combined IgG/IgM sensitivity of 100% for most serotypes.^8^ However, the nucleocapsid protein is highly immunogenic but also exhibits cross-reactivity. Truncated nucleocapsid antigens have improved the serotyping of infections caused by the Sin Nombre virus and ANDV, including sera from the early acute phase with low IgM and IgG titres.^9^

Rapid tests are not merely a suggestion, but an underutilised tool. A 5-min immunochromatographic IgM tests for the Puumala virus have demonstrated high diagnostic performance (100% sensitivity and 99% specificity) (Table 1).^7^ Equally IgM rapid tests for the Puumala, Dobrava and Hantaan viruses also produces results in 5 min, with single-antigen tests showing a sensitivity of 96–100% and a specificity of 96–100% (Table 1).^10^ In Chile, a Puumala-based lateral flow test showed cross-reactivity with ANDV in patients with suspected HCPS and achieved a sensitivity of 97% and a specificity of 90% compared with the capture IgM ELISA, even though it was not Andes-specific.^11^ Available point-of-care approaches using recombinant hantavirus nucleoprotein have demonstrated sensitivities of 88–100% and specificities of 97–100% in human and animal samples studied, supporting the feasibility of simple, field-deployable tests.^12^ These studies underline the notion that even an imperfect rapid serological test can assist with triage in hospitals located far from reference centres, provided that positive or inconclusive results are cross-checked with confirmatory tests.

**Table 1:** Commercially available hantavirus serological assays in the EU, their New World/Andes virus coverage and gaps in rapid on-site diagnostics.

Manufacturer/assay	Format	Antibody class	Virus coverage	New World/ANDV coverage	Potential role	Key limitation
EUROIMMUN Hantavirus Pool 2 “America”	Enzyme-Linked Immunosorbent Assay (ELISA)	IgG/IgM	American hantavirus pool	New World reactive pool assay	Central- laboratory screening	Not rapid; Limited species discrimination
EUROIMMUN Hantavirus Mosaic 3 “America”	Indirect Immunofluorescence Test (IIFT)	IgG/IgM	Sin Nombre; ANDV	Includes ANDV	Differentiated laboratory serology and confirmation	Not rapid; requires specialist expertise
Mikrogen recomLine HantaPlus	Line Immunoassay	IgG/IgM	Puumala; Sin Nombre; Hantaan; Dobrava; Seoul	Includes Sin Nombre; no explicit ANDV antigen	Confirmatory serology and broad antibody detection	Not rapid; no explicit ANDV antigen
Reagena ReaScan + PUUMALA IgM and ReaScan DOBRAVA-HANTAAN IgM	Lateral-flow rapid test	IgM	Puumala; Dobrava; Hantaan	Old World Only	Rapid triage for HFRS/nephropathia epidemica	No New World/ANDV coverage
Abbott Bioline Hantaan Virus Test	Lateral-flow rapid test	IgG/IgM/IgA	Hantaan	Old World Only	Rapid triage for HFRS	No New World/ANDV coverage
Gap: ANDV-specific rapid test	Lateral-flow rapid test	IgG/IgM	ANDV	ANDV	Needed for decentralised outbreak response	Represents a current preparedness gap

Newer diagnostic platforms such as the recombinant nucleocapsid antigen-based point-of-care HANTEC assay was able to detect hantavirus antibodies from humans and rodents with a sensitivity of 87–100% and a specificity of 97–100%,^12^ whilst homogeneous FRET-based immunoassays achieved a sensitivity of 100% and a specificity of 99% in acute Puumala virus infections.^13^ These advances demonstrate that rapid hantavirus serodiagnostics can match the performance of central laboratories.

While molecular assays and sequencing are indispensable for confirmation, they do not eliminate the need for widely available rapid, accessible, reliable serological triage closer to the patient. Serology is also essential beyond acute diagnosis, as it can identify asymptomatic or previously unrecognised infections after antibody development, thereby helping to define transmission routes more accurately and to estimate the true infection fatality rate rather than only the case fatality rate among clinically apparent patients. In the context of passenger repatriation from *MV* Hondius, affected countries should coordinate follow-up, clinical monitoring, risk communication, and reference-laboratory testing to rapidly identify secondary cases without overstating the wider public health threat.

## Contributors

Thirumalaisamy P. Velavan (TPV): Conceptualization, literature curation, investigation, writing—original draft, writing—review and editing; Jonas Schmidt-Chanasit (JSC): Conceptualization, literature curation, investigation, writing—original draft, writing—review and editing. Both authors contributed equally to the development of the commentary, critically reviewed the final draft and approved the submitted version.

## Declaration of interests

The authors declare that they have no known competing financial interests.
